# Viruses and atypical bacteria in the respiratory tract of immunocompromised and immunocompetent patients with airway infection

**DOI:** 10.1007/s10096-020-03878-9

**Published:** 2020-05-27

**Authors:** Maria Reckziegel, Claudia Weber-Osel, Renate Egerer, Bernd Gruhn, Florian Kubek, Mario Walther, Stefanie Wilhelm, Roland Zell, Andi Krumbholz

**Affiliations:** 1 Section of Experimental Virology, Institute of Medical Microbiology, Jena University Hospital, Jena, Germany; 2 Present Address: Department of Hematology/Oncology, Clinic of Internal Medicine II, Jena University Hospital, Jena, Germany; 3 Present Address: Department of Medicine II, Catholic Hospital ‘St. Johann Nepomuk’, Erfurt, Germany; 4 Institute of Medical Microbiology, Jena University Hospital, Jena, Germany; 5 Department of Pediatrics, Jena University Hospital, Jena, Germany; 6 Department of Fundamental Sciences, Jena University of Applied Sciences, Jena, Germany; 7 Institute of Infection Medicine, Christian-Albrechts-Universität zu Kiel and University Medical Center Schleswig-Holstein, Brunswiker Straße 4, D-24105 Kiel, Germany

**Keywords:** Respiratory infection, Immunosuppression, Multiplex PCR, Pathogen spectrum

## Abstract

Respiratory tract infections (RTI) can take a serious course under immunosuppression. Data on the impact of the underlying pathogens are still controversial. Samples from the upper (*n* = 322) and lower RT (*n* = 169) were collected from 136 children and 355 adults; 225 among them have been immunocompromised patients. Exclusion criteria were presence of relevant cultivable microorganisms, C-reactive protein > 20 mg/dl, or procalcitonin > 2.0 ng/ml. Samples were tested by PCR for the presence of herpesviruses (HSV-1/-2; VZV; CMV; HHV6; EBV), adenoviruses, bocaviruses, entero-/rhinoviruses (HRV), parechoviruses, coronaviruses, influenza viruses (IV), parainfluenza viruses as well as for pneumoviruses (HMPV and RSV), and atypical bacteria (*Mycoplasma pneumoniae*, M.p.; *Chlamydia pneumoniae*, C.p.). Viral/bacterial genome equivalents were detected in more than two-thirds of specimens. Under immunosuppression, herpesviruses (EBV 30.9%/14.6%, *p* < 0.001; CMV 19.6%/7.9%, *p* < 0.001; HSV-1: 14.2%/7.1%, *p* = 0.012) were frequently observed, mainly through their reactivation in adults. Immunocompromised adults tended to present a higher RSV prevalence (6.4%/2.4%, *p* = 0.078). Immunocompetent patients were more frequently tested positive for IV (15.0%/5.8%, *p* = 0.001) and M.p. (6.4%/0.4%, *p* < 0.001), probably biased due to the influenza pandemic of 2009 and an M.p. epidemic in 2011. About 41.8% of samples were positive for a single pathogen, and among them EBV (19.9%) was most prevalent followed by HRV (18.2%) and IV (16.6%). HSV-2 and C.p. were not found. Marked seasonal effects were observed for HRV, IV, and RSV. Differences in pathogen prevalence were demonstrated between immunocompetent and immunocompromised patients. The exact contribution of some herpesviruses to the development of RTI remains unclear.

**Electronic supplementary material:**

The online version of this article (10.1007/s10096-020-03878-9) contains supplementary material, which is available to authorized users.

## Introduction

Infections of the upper respiratory tract (URTI) are among the most frequent infections worldwide. These are mainly caused by RNA viruses. Among them, influenza viruses (IV), pneumoviruses (respiratory syncytial virus, RSV; human metapneumovirus, HMPV), but also parainfluenza viruses (PIV), coronaviruses (CoV), and rhinoviruses (HRV) are considered by World Health Organization a global health burden [1]. Serious RTI through respiratory viruses are frequently observed under immunosuppression, for example, in solid organ transplant recipients [2].

The current breakthroughs of immunomodulating therapies in medicine contribute to the continuous increase of patients being under iatrogenic immunosuppression and being at risk for pulmonary infections [3]. In general, suppression of T cell function is associated with a higher susceptibility for infection or reactivation of various viruses [4, 5]. Impairment of Th1-cell activity but also of humoral immunity, both, facilitates the development of viral RTI. In immunocompromised patients, higher morbidity and sometimes also mortality rates through infections, for example, with adenoviruses (AdV), IV, PIV, RSV, HMPV, but also of secondary complications like bacterial pneumonia have been observed [5–17]. Furthermore, particularly transplant patients are at risk for reactivation of diverse herpesviruses (herpes simplex virus-1/-2, HSV-1/-2; varicella zoster virus, VZV; cytomegalovirus, CMV; human herpesvirus 6, HHV-6; Epstein-Barr virus, EBV) [12, 15, 17–20]. Multiple viral infections/reactivations can occur [21] as well as indirect interactions of viruses with bacteria [22–24]. These aspects may challenge interpretation of diagnostic findings. The frequency of viral infections/reactivations is also influenced by factors like the underlying disease, therapeutic regimes, as well as the type of transplant and HLA mismatches [12, 19].

Thus, fast and efficient diagnostic methods that cover a broad spectrum of viruses, bacteria, but also fungi and parasites are in urgent need to deal with the aforementioned challenges. The availability of such methods is of particular importance in stem cell transplant recipients where clinical symptoms of RTI are variable or may be mimicked by graft-versus-host disease [25, 26]. Early diagnosis enables limited antiviral interventions [16, 27] and may prevent further cross-transmission [28]. However, detection of viral genome equivalents does not necessarily mean a causative role of this virus, and particularly immunocompromised patients can shed viruses over a prolonged time period [13, 29, 30]. Furthermore, there is increasing evidence of the existence of a respiratory virome which is defined by the presence of common viral pathogens, rare viruses, and viruses of unknown pathogenicity [31]. Thus, the exact contribution of a single virus to the development of RTI is still controversial.

We tried to consider most of these aspects by performing an observational study addressing the spectrum and impact of respiratory viruses but also of herpesviruses and the atypical bacteria *Mycoplasma pneumoniae* (M.p.) and *Chlamydia pneumoniae* (C.p.) in patients with respiratory symptoms. For this, an underlying infection through relevant cultivable microorganisms was largely ruled out. Data were analyzed with respect to patients’ immune status and age.

## Material and methods

This study included 322 samples from the upper (nasal or throat swabs and washings), and 169 samples from the lower (broncheoalveolar/tracheal washings, induced sputum) respiratory tract (URT/LRT) collected over a period of 40 months beginning in September 2009 to December 2012 from 266 healthy and 225 immunocompromised patients with symptoms of a RTI (i.e., common cold, cough with/without sputum, dyspnea, and fever). This setting included samples from patients with respiratory symptoms under neutropenia or lung-transplant recipients with a recently observed decrease in forced expiratory volume in one second (FEV1). A compromised immune status was defined (i) for solid organ or stem cell transplant recipients under iatrogenic immunosuppression, (ii) in patients with autoimmune disorders under immunosuppressing therapy but also (iii) in cancer patients under chemotherapy/radiation, and (iv) in patients with primary or secondary causes of immunodeficiency including HIV infection.

Samples found to contain relevant cultivable bacteria (*Streptococcus pneumoniae*, *Staphylococcus aureus*, *Haemophilus influenzae*, various Enterobacterales and nonfermenting Gram-negative bacteria, *Mycobacterium tuberculosis*) and fungi were excluded. Furthermore, specimens obtained from patients with a present bacteremia/sepsis were excluded as well as samples from patients with RTI through *Pneumocystis jirovecii* or *Legionella pneumophila*. The oropharyngeal and tracheopulmonal flora was considered if data were available from routine diagnostics. In addition, samples obtained from the LRT of patients with more than 100,000 colonies/ml of oropharyngeal or tracheopulmonal flora, or more than 10,000 colonies/ml of *Enterococcus* spp. or *Candida* spp., were also excluded since such high concentrations may represent an infection rather than colonization. In addition, the presence of procalcitonin (PCT) > 2 ng/ml and/or of C-reactive protein (CRP) > 20 mg/dl in serum leads to exclusion of the RT sample.

The remaining samples were obtained from 99 immunocompetent (median age 1.0 years; 56 males/43 females) and 37 immunocompromised (median age 4.6 years; 18 males/19 females) children and adolescents as well as 167 immunocompetent (median age 46.2 years; 73 males/94 females) and 188 (median age: 57.2 years; 101 males/87 females) immunocompromised adults. Among the immunocompromised cohort, most samples were obtained from patients with hemato-oncological malignancies (28.4%), followed by samples from patients after organ (24.9%) and stem cell (16.4%) transplantation or with autoimmune disorders (13.8%). About 9.8% of samples were included from patients with other conditions of immunosuppression or from solid tumor patients (6.7%).

Specimens were immediately deep frozen (− 80 °C) until nucleic acid extraction. The extraction was done manually with the QIAamp MinElute Virus Spin Kit or automatically with the EZ1 Virus Mini Kit (both QIAGEN, Hilden, Germany). The nucleic acids were stored at − 20 °C and used for synthesis of copy DNA (cDNA) applying the RevertAid H Minus First Strand cDNA Synthesis Kit (ThermoFisher Scientific, Langen, Germany) with random hexamers. Integrity of cDNA was demonstrated by amplification of ß-actin or glyceraldehyde 3-phosphate dehydrogenase (GAPDH) DNA [32, 33] with DreamTaq DNA Polymerase (ThermoFisher Scientific). Presence of herpesviral DNA (HSV-1/-2, VZV, CMV, and HHV-6) was demonstrated by applying conventional PCR [34–41] together with the HotStarTaq DNA polymerase and Q-solution (QIAGEN). Quantitative detection of EBV DNA in samples from the LRT was done in accordance with Krumbholz et al. (2006) [42], but SYBR-green (QuantiTect SYBR Green PCR Kit; QIAGEN) was used instead of hybridization probes. These diagnostic PCRs were continuously approved by successful participation in the External Quality Assurance Service (EQAS) program of Instand e.V. (Düsseldorf, Germany).

For detection of diverse respiratory viruses, all cDNAs were tested with the Seeplex RV5 ACE (covers IV-A; IV-B; RSV-A/-B; AdV; PIV1-3; bocavirus, BoV; HMPV; HRV-A/B; CoV 229E/NL63/OC43/HKU1) and RV12 (covers the same spectrum as RV5, but is not able to detect BoV) ACE Detection Kits. The Seeplex RV15 ACE Detection Kit (includes also PIV-4, HRV-C, and enterovirus detection but is not able to detect CoV HKU1) was used from November 2011, since the distribution of RV5 and RV12 versions was abandoned (all Kits Seegene, Eschborn, Germany). All three multiplex assays have been established in the laboratory using defined EQAS samples from Instand e.V. before testing of study samples. The RV5 Kit is a screening kit and neither allows discrimination between AdV, PIV, and BoV nor between HMPV, HRV, and CoV. To overcome this problem, amplicons were purified after agarose gel-electrophoresis applying the QIAquick Gel Extraction Kit (QIAGEN). Purified DNA was ligated into the pDRIVE cloning vector included in the QIAGEN PCR Cloning Kit, and used for transformation of competent *Escherichia coli* cells. Then, colonies were screened for inserts by PCR applying the DreamTaq DNA Polymerase and oligonucleotides specific for pDRIVE. Amplicons with inserts were purified and sequenced using the DTCS Quick Start Master Mix. Sequence analysis was done on a Beckman CEQ 8000 Genetic Analyzer (all Beckman Coulter, Krefeld, Germany).

For detection of human parechovirus (HPeV) genome equivalents, a semiquantitative real-time PCR was established using cDNA and oligonucleotides [43] together with the QuantiTect SYBR Green PCR Kit (QIAGEN) on a LightCycler 1.5 (Roche, Mannheim, Germany). Positive controls for HPeV-PCR were kindly provided by Dr. Corinna Pietsch and Prof. Dr. Uwe Gerd Liebert (Institute of Virology, University of Leipzig, Germany).

Since enteroviruses were not covered by RV5 and RV12 assays, nearly all samples were screened by a nested PCR protocol detecting a conserved sequence of the 5′-nontranslated region (5′-NTR) [44]. Then, rough-typing was done by sequence analysis of purified PCR products. Detection of HRV was performed by nested amplification of the VP4/2-encoding region [45].

Parallel testing for M.p. and C.p. was done by applying the Diagenode *Mycoplasma pneumoniae* and *Chlamydophila pneumoniae* Kit (R-DiaMCpn, Diagenode s. a., Liège, Belgium) on an ABI7500 real-time PCR system (ThermoFisher Scientific).

Sequence data were analyzed using MEGA 6.0 [46]. All other data were analyzed applying the two-sided Fisher’s exact test implemented in IBM® SPSS® Statistics 20. A *p* value < 0.05 was considered statistically significant.

## Results

This study included 491 samples from immunocompromised or immunocompetent patients with symptoms of RTI collected over a period of 40 months. All samples tested positive for the presence of GAPDH and/or ß-actin. Thus, quality of sampling and nucleic acid extraction was demonstrated (data not shown).

Among the overall study population, genome equivalents of EBV were most frequently detected (22.3%, 84/377), followed by HHV-6 (20.3%, 32/158), HRV (14.1%, 69/491), CMV (13.2%, 65/491), RSV (11.2%, 55/491), and IV (10.8%, 53/491). Genome equivalents of HSV-2 and C.p. were generally not detected (Table 1).

**Table 1:** Prevalence of respiratory viruses, herpesviruses, and atypical bacteria C.p. and M.p. in the upper and lower respiratory tract (URT/LRT) of immunocompromised and immunocompetent patients. The pathogens are listed in alphabetical order

	Immunocompromised patients			Immunocompetent patients		
	Total	URT	LRT	Total	URT	LRT
(a) Overall study population						
Respiratory viruses						
AdV	3/225 (1.3%)	1/110 (0.9%)	2/115 (1.7%)	5/266 (1.9%)	4/212 (1.9%)	1/54 (1.9%)
BoV	4/225 (1.8%)	3/110 (2.7%)	1/115 (0.9%)	8/266 (3.0%)	8/212 (3.8%)	0/54 (0.0%)
CoV	6/225 (2.7%)	4/110 (3.6%)	2/115 (1.7%)	9/266 (3.4%)	8/212 (3.8%)	1/54 (1.9%)
EV	2/222 (0.9%)	1/109 (0.9%)	1/113 (0.9%)	8/264 (3.0%)	8/210 (3.8%)	0/54 (0.0%)
HMPV	4/225 (1.8%)	3/110 (2.7%)	1/115 (0.9%)	7/266 (2.6%)	7/212 (3.3%)	0/54 (0.0%)
HPeV	0/222 (0.0%)	0/109 (0.0%)	0/113 (0.0%)	1/264 (0.4%)	1/210 (0.5%)	0/54 (0.0%)
HRV	30/225 (13.3%)	15/110 (13.6%)	15/115 (13.0%)	39/266 (14.7%)	35/212 (16.5%)	4/54 (7.4%)
IV	13/225 (5.8%)^d^	11/110 (10.0%)^a^	2/115 (1.7%)	40/266 (15.0%)^d^	40/212 (18.9%)^a^	0/54 (0.0%)
PIV	7/225 (3.1%)	4/110 (3.6%)	3/115 (2.6%)	5/266 (1.9%)	5/212 (2.4%)	0/54 (0.0%)
RSV	20/225 (8.9%)	14/110 (12.7%)	6/115 (5.2%)	35/266 (13.2%)	31/212 (14.6%)	4/54 (7.4%)
Herpesviruses						
CMV	44/225 (19.6%)^d^	13/110 (11.8%)	31/115 (27.0%)^b^	21/266 (7.9%)^d^	15/212 (7.1%)	6/54 (11.1%)^b^
EBV	55/178 (30.9%)^d^	18/65 (27.7%)^c^	37/113 (32.7%)	29/199 (14.6%)^d^	17/147 (11.6%)^c^	12/52 (23.1%)
HHV-6	15/71 (21.1%)	6/23 (26.1%)	9/48 (18.8%)	17/87 (19.5%)	10/58 (17.2%)	7/29 (24.1%)
HSV-1	32/225 (14.2%)^b^	16/110 (14.5%)^b^	16/115 (13.9%)	19/266 (7.1%)^b^	14/212 (6.6%)^b^	5/54 (9.3%)
HSV-2	0/225 (0.0%)	0/110 (0.0%)	0/115 (0.0%)	0/266 (0.0%)	0/212 (0.0%)	0/54 (0.0%)
VZV	0/83 (0.0%)	0/42 (0.0%)	0/41 (0.0%)	1/107 (0.9%)	1/88 (1.1%)	0/19 (0.0%)
Atypical bacteria						
C.p.	0/225 (0.0%)	0/110 (0.0%)	0/115 (0.0%)	0/266 (0.0%)	0/212 (0.0%)	0/54 (0.0%)
M.p.	1/225 (0.4%)^d^	0/110 (0.0%)^b^	1/115 (0.9%)^c^	17/266 (6.4%)^d^	11/212 (5.2%)^b^	6/54 (11.1%)^c^
None of these pathogens						
	75/225 (33.3%)	–	–	82/266 (30.8%)	–	–
(b) Children						
Respiratory viruses						
AdV	0/37 (0.0%)	0/35 (0.0%)	0/2 (0.0%)	4/99 (4.0%)	3/95 (3.2%)	1/4 (25.0%)
BoV	2/37 (5.4%)	2/35 (5.7%)	0/2 (0.0%)	7/99 (7.1%)	7/95 (7.4%)	0/4 (0.0%)
CoV	2/37 (5.4%)	1/35 (2.9%)	1/2 (50.0%)	6/99 (6.1%)	6/95 (6.3%)	0/4 (0.0%)
EV	0/37 (0.0%)	0/35 (0.0%)	0/2 (0.0%)	6/98 (6.1%)	6/94 (6.4%)	0/4 (0.0%)
HMPV	2/37 (5.4%)	2/35 (5.7%)	0/2 (0.0%)	4/99 (4.0%)	4/95 (4.2%)	0/4 (0.0%)
HPeV	0/37 (0.0%)	0/35 (0.0%)	0/2 (0.0%)	1/98 (1.0%)	1/94 (1.1%)	0/4 (0.0%)
HRV	9/37 (24.3%)	9/35 (25.7%)	0/2 (0.0%)	26/99 (26.3%)	25/95 (26.3%)	1/4 (25.0%)
IV	2/37 (5.4%)	2/35 (5.7%)	0/2 (0.0%)	12/99 (12.1%)	12/95 (12.6%)	0/4 (0.0%)
PIV	1/37 (2.7%)	1/35 (2.9%)	0/2 (0.0%)	2/99 (2.0%)	2/95 (2.1%)	0/4 (0.0%)
RSV	8/37 (21.6%)	8/35 (22.9%)	0/2 (0.0%)	31/99 (31.3%)	29/95 (30.5%)	2/4 (50.0%)
Herpesviruses						
CMV	0/37 (0.0%)^b^	0/35 (0.0%)^b^	0/2 (0.0%)	11/99 (11.1%)^b^	11/95 (11.6%)^b^	0/4 (0.0%)
EBV	0/16 (0.0%)	0/14 (0.0%)	0/2 (0.0%)	2/73 (2.7%)	2/70 (2.9%)	0/3 (0.0%)
HHV-6	0/5 (0.0%)	0/3 (0.0%)	0/2 (0.0%)	6/35 (17.1%)	6/33 (18.2%)	0/2 (0.0%)
HSV-1	1/37 (2.7%)	1/35 (2.9%)	0/2 (0.0%)	2/99 (2.0%)	2/95 (2.1%)	0/4 (0.0%)
HSV-2	0/37 (0.0%)	0/35 (0.0%)	0/2 (0.0%)	0/99 (0.0%)	0/95 (0.0%)	0/4 (0.0%)
VZV	0/11 (0.0%)	0/11 (0.0%)	–	0/37 (0.0%)	0/37 (0.0%)	–
Atypical bacteria						
C.p.	0/37 (0.0%)	0/35 (0.0%)	0/2 (0.0%)	0/99 (0.0%)	0/95 (0.0%)	0/4 (0.0%)
M.p.	0/37 (0.0%)	0/35 (0.0%)	0/2 (0.0%)	1/99 (1.0%)	1/95 (1.1%)	0/4 (0.0%)
None of these pathogens						
	17/37 (45.9%)^d^	–	–	18/99 (18.2%)^d^	–	–
(c) Adults						
Respiratory viruses						
AdV	3/188 (1.6%)	1/75 (1.3%)	2/113 (1.8%)	1/167 (0.6%)	1/117 (0.9%)	0/50 (0.0%)
BoV	2/188 (1.1%)	1/75 (1.3%)	1/113 (0.9%)	1/167 (0.6%)	1/117 (0.9%)	0/50 (0.0%)
CoV	4/188 (2.1%)	3/75 (4.0%)	1/113 (0.9%)	3/167 (1.8%)	2/117 (1.7%)	1/50 (2.0%)
EV	2/185 (1.1%)	1/74 (1.4%)	1/111 (0.9%)	2/166 (1.2%)	2/116 (1.7%)	0/50 (0.0%)
HMPV	2/188 (1.1%)	1/75 (1.3%)	1/113 (0.9%)	3/167 (1.8%)	3/117 (2.6%)	0/50 (0.0%)
HPeV	0/185 (0.0%)	0/74 (0.0%)	0/111 (0.0%)	0/166 (0.0%)	0/116 (0.0%)	0/50 (0.0%)
HRV	21/188 (11.2%)	6/75 (8.0%)	15/113 (13.3%)	13/167 (7.8%)	10/117 (8.5%)	3/50 (6.0%)
IV	11/188 (5.9%)^c^	9/75 (12.0%)^a^	2/113 (1.8%)	28/167 (16.8%)^c^	28/117 (23.9)^a^	0/50 (0.0%)
PIV	6/188 (3.2%)	3/75 (4.0%)	3/113 (2.7%)	3/167 (1.8%)	3/117 (2.6%)	0/50 (0.0%)
RSV	12/188 (6.4%)^a^	6/75 (8.0%)^a^	6/113 (5.3%)	4/167 (2.4%)^a^	2/117 (1.7%)^a^	2/50 (4.0%)
Herpesviruses						
CMV	44/188 (23.4%)^d^	13/75 (17.3%)^c^	31/113 (27.4%)^b^	10/167 (6.0%)^d^	4/117 (3.4%)^c^	6/50 (12.0%)^b^
EBV	55/162 (34.0%)^b^	18/51 (35.3%)^a^	37/111 (33.3%)	27/126 (21.4%)^b^	15/77 (19.5%)^a^	12/49 (24.5%)
HHV-6	15/66 (22.7%)	6/20 (30.0%)	9/46 (19.6%)	11/52 (21.2%)	4/25 (16.0%)	7/27 (25.9%)
HSV-1	31/188 (16.5%)^a^	15/75 (20.0%)^a^	16/113 (14.2%)	17/167 (10.2%)^a^	12/117 (10.3%)^a^	5/50 (10.0%)
HSV-2	0/188 (0.0%)	0/75 (0.0%)	0/113 (0.0%)	0/167 (0.0%)	0/117 (0.0%)	0/50 (0.0%)
VZV	0/72 (0.0%)	0/31 (0.0%)	0/41 (0.0%)	1/70 (1.4%)	1/51 (2.0%)	0/19 (0.0%)
Atypical bacteria						
C.p.	0/188 (0.0%)	0/75 (0.0%)	0/113 (0.0%)	0/167 (0.0%)	0/117 (0.0%)	0/50 (0.0%)
M.p.	1/188 (0.5%)^d^	0/75 (0.0%)^c^	1/113 (0.9%)^c^	16/167 (9.6%)^d^	10/117 (8.5%)^c^	6/50 (12.0%)^c^
None of these pathogens						
	58/188 (30.6%)	–	–	64/167 (38.3%)	–	–

Four hundred eighty-six of the 491 samples were tested for EV/HPeV, 377 for EBV, 190 for VZV, and 158 for the presence of HHV-6 DNA, respectively

*AdV* adenovirus, *BoV* bocavirus, *CMV* cytomegalovirus, *CoV* coronavirus, *C*.*p*. *Chlamydia pneumoniae*, *EBV* Epstein-Barr virus, *EV* enterovirus, *HHV*-*6* human herpesvirus 6, *HMPV* human metapneumovirus, *HPeV* human parechovirus, *HRV* human rhinovirus, *HSV*-*1* herpes simplex virus 1, *HSV*-*2* herpes simplex virus 2, *M*.*p*. *Mycoplasma pneumoniae*, *PIV* influenza virus, *PIV* parainfluenza virus, *RSV* respiratory syncytial virus, *VZV* varicella-zoster virus

^a^Level of significance up to 10%

^b^Level of significance up to 5%

^c^Level of significance up to 1%

^d^Level of significance up to ≤ 0.1%

**Fig. 1:**
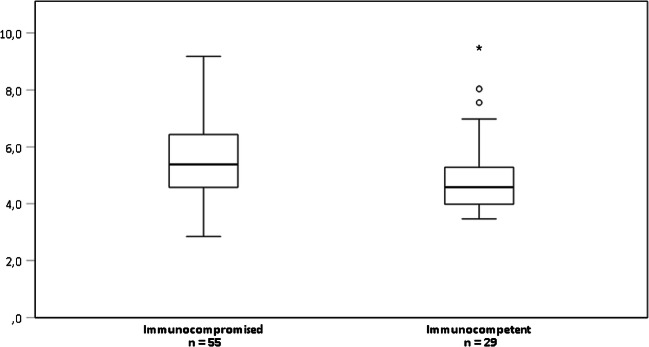
Comparison of EBV-DNA copies/ml in respiratory specimens from immunocompromised and immunocompetent patients. Data are presented in a logarithmic scale. The median EBV concentration is significantly higher in immunocompromised patients (*p* = 0.030, Mann-Whitney *U* test)

With respect to patients’ immune status, DNA of EBV (30.9% vs. 14.6%), CMV (19.6% vs. 7.9%), and HSV-1 (14.2% vs. 7.1%) was significantly more prevalent in immunocompromised patients while genome equivalents of IV (5.8% vs. 15.0%) or M.p. (0.4% vs. 6.4%) were more frequently observed in their immunocompetent counterpart. The higher prevalence of CMV and EBV was only observed in immunocompromised adults (23.4% vs. 6.0% and 34.0% vs. 21.4%, respectively). Moreover, this patient group tended to present a higher prevalence of HSV-1 (16.5% vs. 10.2%) and RSV (6.4% vs. 2.4%) (Table 1). The median concentration of EBV DNA in the LRT was significantly higher in immunocompromised patients. Moreover, this group presented a higher prevalence of EBV concentration exceeding 100,000 copies/ml (Fig. 1, Supplementary Table 1). Roughly one-third (35.7%, 30/84) of all EBV genome detections were not associated with other pathogens, while two-thirds of EBV-positive samples revealed double (42.9%, 36/84) or multiple (21.4%, 18/84) detections together with other viruses (Supplementary Fig. 1).

In 26.3% (129/491) of samples found to be pathogen-positive, multiple agents were detected. Among them were samples with two (20.4%), three (5.1%), four (0.6%), or even five (0.2%) different viruses/bacteria (Supplementary Fig. 1). The combination of two herpesviruses (HHV-6/EBV 13.2%, 5/38; CMV/EBV 9.6%, 8/83; CMV/HSV-1 8.0%, 8/100) but also of EBV and IV (6.0%, 5/83) or M.p. (6.0%, 5/83) as well as of RSV and HRV (5.0%, 5/100) was frequently observed. Bocaviral DNA was found together with other viruses/M.p. (74.9%, 9/12). However, in younger children (≤ 2 years), monoinfections through BoV were observed (Table 2, Supplementary Fig. 1).

**Table 2:**
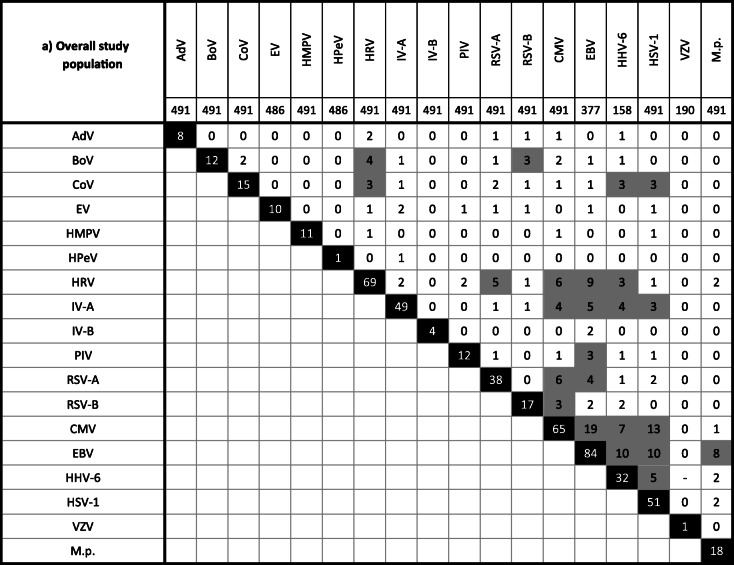

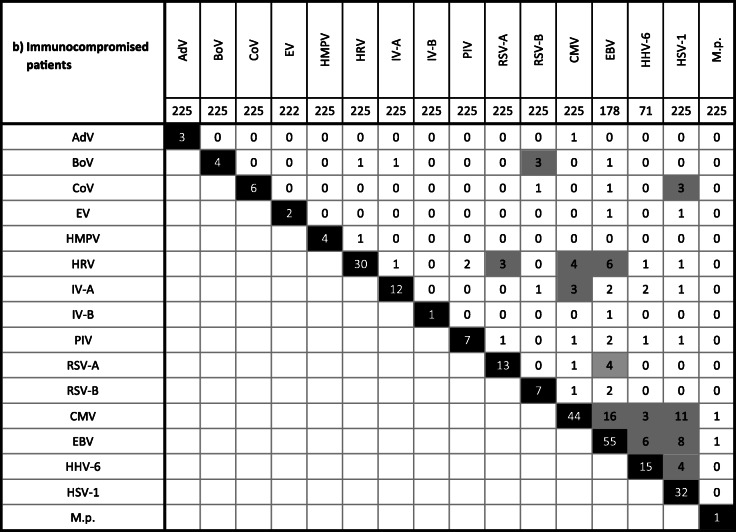

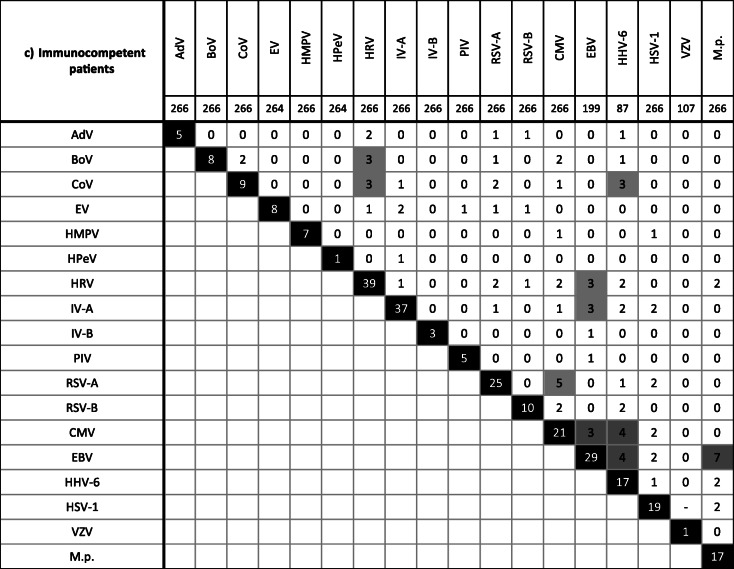
Detection of multiple pathogens in the respiratory tract of the overall study population (a) as well as of immunocompromised (b) and immunocompetent (c) patients. The gray boxes indicate frequent co-infections. Note that due to multiple detection (i.e., more than two pathogens), the sum of the frequencies given in these boxes may be higher the total frequency given in the black box. See also Suppl. Figure 1

Significant seasonal effects were recorded in the immunocompetent group for HRV with a high prevalence in autumn and for IV with an increased prevalence in winter. Seasonal effect was significant in both patient groups for RSV with increased prevalence in winter and spring (Supplementary Table 2). Interestingly, slight seasonality was also observed for HHV-6 in the immunocompetent group. In addition, some interannual variation was found for M.p. and IV (data not shown), which was most likely associated to the 2009-influenza pandemic and an M.p. epidemic in 2011, respectively.

## Discussion

In this monocentric study, genome equivalents of viruses and M.p. were frequently detected in immunocompromised (66.7%) and immunocompetent (69.2%) patients with respiratory symptoms (Table 1).

Since a contribution of relevant cultivable microorganisms to patient symptoms was largely excluded, a causative role of the pathogens detected in this study has to be considered. Previously, a comparable approach was used to identify viral causes of severe RTI in children [47]. The stringent exclusion criteria may account for the low number of patient samples included in this study and may have neglected possible additive or synergistic effects between bacteria, fungi, and viruses.

In particular, we found a high prevalence of herpesviruses in immunocompromised adults with respiratory infections. Nearly one-third of them was tested positive for EBV and every fourth patient presented CMV in his respiratory tract. In children, herpesviral DNA was rarely detected which reflects the generally increasing infestation rate observed over life-time [48–52] and indicates viral reactivation as a major cause for pathogen detection. The higher prevalence of EBV, CMV, and HSV-1 in the airways of adults was associated with the state of immunosuppression. This is in line with the fact that herpesviral reactivation is facilitated by the impaired immune system [53]. It is still controversial whether this reactivation contributes to respiratory pathology or just represents an indicator of excessive immunosuppression. For CMV, however, there is no doubt that this betaherpesvirus is responsible for LRTI in immunocompromised patients [17, 20]. CMV pneumonia is considered as likely when viral DNA has been detected in BAL of symptomatic patients [17]. Thus, in our study, a remarkable proportion of immunocompromised adults revealed signs of suspected CMV pneumonia (Table 1) and may benefit from antiviral prophylaxis or therapy.

While HSV-1 DNA was slightly more prevalent in the URT of immunocompromised patients, we found nearly comparable detection rates in the LRT of both patients groups (Table 1). This is in line with a previous study [54]. Interestingly, the same authors found that higher HSV-1 concentrations were associated with a poor patient outcome [54]. As for CMV, definitive diagnosis of HSV-1 pneumonitis depends on the presence of viral antigen within the LRT tissues [17].

In contrast to a recent report [55], we found two-times higher EBV DNA prevalence in immunocompromised patients compared to their immunocompetent counterparts (Table 1). Previously, EBV DNA was frequently detected in patients with pneumonia, respiratory insufficiency, and other bronchopneumopathies, but its presence was not associated with increased 28-day mortality [55]. In addition, the same authors reported no difference in EBV concentration between immunocompromised and immunocompetent patients [55], which is in contrast to our findings (Fig. 1, Supplementary Table 1). Nevertheless, the contribution of EBV to the development of respiratory symptoms is still controversially discussed in the literature and remains unclear so far [55–57]. There is, however, some evidence that EBV reactivation—like that of other herpesviruses—may trigger inflammation which is associated to transplant rejection or interstitial lung disease [20, 58–60].

HHV-6 DNA was found at similar high frequencies of ca. 20% in both patient collectives. Interpretation of our results, however, is limited since our PCR protocol may have also detected chromosomally integrated viral DNA [61] and cannot differentiate between HHV-6A and HHV-6B. The latter variant is more commonly implicated in human disease [62]. Moreover, HHV-6 was frequently observed in combination with other herpesviruses (Table 2) as also seen by others [62]. Thus, the contribution of HHV-6 to RTI remains unclear.

Other herpesviruses (VZV, HSV-2) were found to be negligible in this study (Table 1) which is in line with the literature [17, 20, 54, 63].

Most of our results were obtained by end-point PCR. The consideration of viral concentration—as it is exemplarily shown here for EBV—may be useful in order to better unravel the contribution of herpesviruses to the development of lung pathology [20]. The observed frequencies of respiratory viruses were comparable to data from the German Laboratory Network (https://clinical-virology.net/en/charts/chart/ctype/count/network/resp/section/viruses) and to another study from Germany [64]. Genome equivalents of RSV and HRV were prevalent in children while HRV and IV were frequent in adults. Interestingly, immunocompromised adults tended to have a higher prevalence of RSV (Table 1). This supports previous data on the contribution of RSV to morbidity and mortality in this patient group [65].

There were various examples of single detections, which are probably indicative for infection, but also of co-presence of two or more pathogens (Table 2, Supplementary Fig. 1). Bocaviral DNA, for instance, was frequently found in combination together with further viral genomes as also reported by others [66]. In children of 2 years and younger, however, this parvovirus was detected solely. Previously, isolated BoV infection was shown to be a likely cause of severe acute RTI in children [67]. In a German study, BoV DNA was demonstrated in 10.3% of nasal swabs obtained from children with respiratory symptoms [68]. This prevalence is largely comparable to our results. Same authors indicated a mean age of 1.8 years for BoV detection. In 39.1%, bocaviral DNA was detected together with other pathogens [68].

Interestingly, analysis of the EV 5′-NTR sequences gave some evidence for the presence of EV-D68 in the airways of three adults and one toddler. EV-D68 infection is associated with the development of acute flaccid myelitis and severe respiratory illness [69].

Parechoviral RNA was found only in a 2-year-old immunocompetent child with IA-V infection. Human parechoviruses can cause mild gastrointestinal and respiratory disease but also sepsis-like illness and meningitis in infants [70]. The general low prevalence of HPeV in this study is in line with a previous report [64].

The possible etiology of RTI was not clarified in 45.9% of immunocompromised children (Table 1). Under these conditions, application of broad diagnostic technologies like next-generation sequencing could be useful in identification of the underlying pathogen [71]. Moreover, CMV detection rate in samples from the URT of immunocompetent children was surprisingly high (Table 1).

Seasonal effects were evident for several respiratory viruses (Supplementary Table 2). Slight seasonality was also observed for HHV-6 in immunocompetent patients. Interpretation of this finding, however, is unclear. The high prevalence of M.p. in 2011 may be explained by an epidemic observed in Germany [72]. In line with this, the 2009 pandemic caused by A/H1N1pdm09 may account for a further study bias.

DNA of C.p. was generally not detected in our study. This is in line with the low prevalence of 0.2% reported recently [73], but also with data from the Respiratory Viruses Network (https://clinical-virology.net/en/charts/chart/ctype/count/network/resp/section/bacteria). It is hypothesized that the prevalence of C.p. was overestimated in previous reports, most likely due to usage of nested-PCR methods or inclusion of serological data. Previously, both C.p. and M.p. were found to be not relevant in critically ill patients with hospital-acquired respiratory tract infections [74].

In summary, with PCR, we found a high prevalence of viral pathogens in the respiratory airways of immunocompetent and immunocompromised patients. In addition, we demonstrated co-presence of several viruses, presumably due to reactivation of herpesviruses.

## Electronic supplementary material

ESM 1 (DOCX 281 kb).
