# Correction to: Viruses and atypical bacteria in the respiratory tract of immunocompromised and immunocompetent patients with airway infection

**DOI:** 10.1007/s10096-021-04367-3

**Published:** 2021-12-01

**Authors:** Maria Reckziegel, Claudia Weber-Osel, Renate Egerer, Bernd Gruhn, Florian Kubek, Mario Walther, Stefanie Wilhelm, Roland Zell, Andi Krumbholz

**Affiliations:** 1 Section of Experimental Virology, Institute of Medical Microbiology, Jena University Hospital, Jena, Germany; 2 Present Address: Department of Hematology/Oncology, Clinic of Internal Medicine II, Jena University Hospital, Jena, Germany; 3 Present Address: Department of Medicine II, Catholic Hospital ‘St. Johann Nepomuk’, Erfurt, Germany; 4 Institute of Medical Microbiology, Jena University Hospital, Jena, Germany; 5 Department of Pediatrics, Jena University Hospital, Jena, Germany; 6 Department of Fundamental Sciences, Jena University of Applied Sciences, Jena, Germany; 7 Institute of Infection Medicine, Christian-Albrechts-Universität zu Kiel and University Medical Center Schleswig-Holstein, Brunswiker Straße 4, 24105 Kiel, Germany

**Correction to: European Journal of Clinical Microbiology & Infectious Diseases (2020) 39:1581–1592**

10.1007/s10096-020-03878-9

The article “Viruses and atypical bacteria in the respiratory tract of immunocompromised and immunocompetent patients with airway infection”, written by Maria Reckziegel, Claudia Weber-Osel, Renate Egerer, Bernd Gruhn, Florian Kubek, Mario Walther, Stefanie Wilhelm, Roland Zell, and Andi Krumbholz, was originally published Online First without Open Access. After publication in volume 39, issue 8, pages 1581–1592 the author decided to opt for Open Choice and to make the article an Open Access publication. Therefore, the copyright of the article has been changed to © The Author(s) 2020 and the article is forthwith distributed under the terms of the Creative Commons Attribution 4.0 International License, which permits use, sharing, adaptation, distribution and reproduction in any medium or format, as long as you give appropriate credit to the original author(s) and the source, provide a link to the Creative Commons licence, and indicate if changes were made. The images or other third party material in this article are included in the article's Creative Commons licence, unless indicated otherwise in a credit line to the material. If material is not included in the article's Creative Commons licence and your intended use is not permitted by statutory regulation or exceeds the permitted use, you will need to obtain permission directly from the copyright holder. To view a copy of this licence, visit http://creativecommons.org/licenses/by/4.0/.

The original article has been corrected.

