# Supplementary material for: Viruses and atypical bacteria in the respiratory tract of immunocompromised and immunocompetent patients with airway infection
Source: Eur J Clin Microbiol Infect Dis. 2020 May 27;39(8):1581–92. doi: 10.1007/s10096-020-03878-9 (PMC7253234; doi:10.1007/s10096-020-03878-9)
Supplement: Supplementary file 1 — (DOCX 281 kb). [file 10096_2020_3878_MOESM1_ESM.docx]

Supplementary material

**Viruses and atypical bacteria in the respiratory tract of immunocompromised and immunocompetent patients with airway infection**

Maria Reckziegel^1a#^, Claudia Weber-Osel^1b#^, Renate Egerer^2^, Bernd Gruhn^3^, Florian Kubek^1^, Mario Walther^4^, Stefanie Wilhelm^1^, Roland Zell^1#^, Andi Krumbholz^5#^

1 Section of Experimental Virology, Institute for Medical Microbiology, Jena University Hospital, Jena, Germany

2 Institute of Medical Microbiology, Jena University Hospital, Jena, Germany

3 Department of Pediatrics, Jena University Hospital, Jena, Germany

4 Jena University of Applied Sciences, Department of Fundamental Sciences, Jena, Germany

5 Institute of Infection Medicine, Christian-Albrechts-Universität zu Kiel and University Medical Center Schleswig-Holstein, Kiel, Germany

a present address: Department of Hematology/Oncology, Clinic of Internal Medicine II, Jena University Hospital, Jena, Germany

b present address: Department of Medicine II, Catholic Hospital 'St. Johann Nepomuk', Erfurt, Germany

# these first and senior authors contributed equally to this study

* corresponding author: Andi Krumbholz, M.D.

Institute of Infection Medicine, Christian-Albrechts-Universität zu Kiel and University Medical Center Schleswig-Holstein

Brunswiker Straße 4

D-24105 Kiel

Germany

krumbholz@infmed.uni-kiel.de

Tel: +49-431-220-10-131

Keywords: Respiratory infection, immunosuppression, multiplex PCR, pathogen spectrum

**Supplementary Table 1:** Quantification of EBV DNA concentration in respiratory tract samples from immunocompromised and immunocompetent patients

| **EBV concentration (copies/ml)** | **Immunocompromised**  **patients** | **Immunocompetent**  **patients** | **overall study popoulation** | ***p*-value** |
| --- | --- | --- | --- | --- |
| < 10,000 | 8 (14.5 %) | 8 (27.6 %) | 16 (19.0 %) | 0.158 |
| ≥ 10,000 - ≤ 100,000 | 12 (21.8 %) | 12 (41.4 %) | 24 (28.6 %) | 0.077 |
| > 100,000 | 35 (63.6 %) | 9 (31.0 %) | 44 (52.4 %) | **0.006** |
| total | 55 | 29 | 84 |  |

**Supplementary Table 2.** Seasonality in the frequency of pathogen detection. Significant differences are marked by an asterisk in the total-column for the respective pathogen. Evaluations are based on Fisher’s exact test.

**a) Overall study population**:

|  | **Immunocompromised patients** | | | | | **Immunocompetent patients** | | | | | |
| --- | --- | --- | --- | --- | --- | --- | --- | --- | --- | --- | --- |
|  | **spring**  **(Mar-May)** | **summer**  **(Jun-Aug)** | **autumn**  **(Sep-Nov)** | **winter**  **(Dec-Feb)** | **total** | **spring**  **(Mar-May)** | **summer**  **(Jun-Aug)** | **autumn**  **(Sep-Nov)** | **winter**  **(Dec-Feb)** | **total** | |
| ***Respiratory viruses*** | | | | | | | | | | | |
| AdV | 2 (3.4%) | 0 (0.0%) | 1 (1.6%) | 0 (0.0%) | 3 | 3 (4.2%) | 0 (0.0%) | 0 (0.0%) | 2 (1.8%) | 5 | |
| BoV | 1 (1.7%) | 0 (0.0%) | 0 (0.0%) | 3 (4.4%) | 4 | 3 (4.2%) | 2 (6.5%) | 1 (2.0%) | 2 (1.8%) | 8 | |
| CoV | 2 (3.4%) | 0 (0.0%) | 0 (0.0%) | 4 (5.9%) | 6 | 2 (2.8%) | 0 (0.0%) | 2 (4.0%) | 5 (4.4%) | 9 | |
| EV | 1 (1.7%) | 0 (0.0%) | 1 (1.6%) | 0 (0.0%) | 2 | 0 (0.0%) | 1 (3.2%) | 3 (6.0%) | 4 (3.5%) | 8 | |
| HMPV | 0 (0.0%) | 0 (0.0%) | 0 (0.0%) | 4 (5.9%) | 4* | 2 (2.8%) | 1 (3.2%) | 0 (0.0%) | 4 (3.5%) | 7 | |
| HPeV | 0 (0.0%) | 0 (0.0%) | 0 (0.0%) | 0 (0.0%) | 0 | 0 (0.0%) | 0 (0.0%) | 1 (2.0%) | 0 (0.0%) | 1 | |
| HRV | 6 (10.2%) | 4 (11.1%) | 13 (21.0%) | 7 (10.3%) | 30 | 10 (14.1%) | 3 (9.7%) | 14 (28.0%) | 12 (10.53%) | 39* | |
| IV | 5 (8.5%) | 0 (0.0%) | 2 (3.2%) | 6 (8.8%) | 13 | 7 (9.9%) | 0 (0.0%) | 8 (16.0%) | 25 (21.9%) | 40* | |
| PiV | 3 (5.1%) | 1 (2.8%) | 2 (3.2%) | 1 (1.5%) | 7 | 3 (4.2%) | 1 (3.2%) | 1 (2.0%) | 0 (0.0%) | 5 | |
| RSV | 7 (11.9%) | 1 (2.8%) | 1 (1.6%) | 11 (16.2%) | 20** | 9 (12.7%) | 1 (3.2%) | 1 (2.0%) | 24 (21.1%) | 35** | |
| ***Herpesviruses*** | | | | | | | | | | | |
| CMV | 8 (13.6%) | 7 (19.4%) | 14 (22.6%) | 15 (22.1%) | 44 | 6 (8.5%) | 3 (9.7%) | 3 (6.0%) | 9 (7.9%) | 21 | |
| EBV | 17 (34.0%) | 9 (30.0%) | 14 (29.2%) | 15 (30.0%) | 55 | 8 (14.5%) | 6 (24.0%) | 4 (13.8%) | 11 (12.2%) | 29 | |
| HHV-6 | 5 (38.5%) | 3 (21.4%) | 5 (20.0%) | 2 (10.5%) | 15 | 6 (26.1%) | 1 (6.7%) | 0 (0.0%) | 10 (29.4%) | 17* | |
| HSV-1 | 13 (22.0%) | 5 (13.9%) | 5 (8.1%) | 9 (13.2%) | 32 | 6 (8.5%) | 3 (9.7%) | 2 (4.0%) | 8 (7.0%) | 19 | |
| VZV | 0 (0.0%) | 0 (0.0%) | 0 (0.0%) | 0 (0.0%) | 0 | 0 (0.0%) | 0 (0.0%) | 0 (0.0%) | 1 (1.8%) | 1 | |
| ***Atypical bacteria*** | | | | | | | | | | | |
| M.p. | 0 (0.0%) | 0 (0.0%) | 0 (0.0%) | 1 (1.5%) | 1 | 5 (7.0%) | 3 (9.7%) | 6 (12.0%) | 3 (2.6%) | | 17 |

**b) Children:**

|  | **Immunocompromised patients** | | | | | **Immunocompetent patients** | | | | |
| --- | --- | --- | --- | --- | --- | --- | --- | --- | --- | --- |
|  | **spring**  **(Mar-May)** | **summer**  **(Jun-Aug)** | **autumn**  **(Sep-Nov)** | **winter**  **(Dec-Feb)** | **total** | **spring**  **(Mar-May)** | **summer**  **(Jun-Aug)** | **autumn**  **(Sep-Nov)** | **winter**  **(Dec -Feb)** | **total** |
| ***Respiratory viruses*** | | | | | | | | | | |
| AdV | 0 (0.0%) | 0 (0.0%) | 0 (0.0%) | 0 (0.0%) | 0 | 3 (11.5%) | 0 (0.0%) | 0 (0.0%) | 1 (2.1%) | 4 |
| BoV | 0 (0.0%) | 0 (0.0%) | 0 (0.0%) | 2 (10.5%) | 2 | 3 (11.5%) | 2 (22.2%) | 1 (6.3%) | 1 (2.1%) | 7 |
| CoV | 0 (0.0%) | 0 (0.0%) | 0 (0.0%) | 2 (10.5%) | 2 | 1 (3.8%) | 0 (0.0%) | 1 (6.3%) | 4 (8.3%) | 6 |
| EV | 0 (0.0%) | 0 (0.0%) | 0 (0.0%) | 0 (0.0%) | 0 | 0 (0.0%) | 1 (11.1%) | 2 (12.5%) | 3 (6.3%) | 6 |
| HMPV | 0 (0.0%) | 0 (0.0%) | 0 (0.0%) | 2 (10.5%) | 2 | 0 (0.0%) | 1 (11.1%) | 0 (0.0%) | 3 (6.3%) | 4 |
| HPeV | 0 (0.0%) | 0 (0.0%) | 0 (0.0%) | 0 (0.0%) | 0 | 0 (0.0%) | 0 (0.0%) | 1 (6.3%) | 0 (0.0%) | 1 |
| HRV | 2 (40.0%) | 0 (0.0%) | 3 (30.0%) | 4 (21.1%) | 9 | 8 (30.8%) | 1 (11.1%) | 9 (56.3%) | 8 (16.7%) | 26* |
| IV | 0 (0.0%) | 0 (0.0%) | 1 (10.0%) | 1 (5.3%) | 2 | 2 (7.7%) | 0 (0.0%) | 3 (18.8%) | 7 (14.6%) | 12 |
| PiV | 1 (20.0%) | 0 (0.0%) | 0 (0.0%) | 0 (0.0%) | 1 | 1 (3.8%) | 1 (11.1%) | 0 (0.0%) | 0 (0.0%) | 2 |
| RSV | 2 (40.0%) | 0 (0.0%) | 1 (10.0%) | 5 (26.3%) | 8 | 9 (34.6%) | 1 (11.1%) | 1 (6.3%) | 20 (41.7%) | 31* |
| ***Herpesviruses*** | | | | | | | | | | |
| CMV | 0 (0.0%) | 0 (0.0%) | 0 (0.0%) | 0 (0.0%) | 0 | 3 (11.5%) | 1 (11.1%) | 1 (6.3%) | 6 (12.5%) | 11 |
| EBV | 0 (0.0%) | 0 (0.0%) | 0 (0.0%) | 0 (0.0%) | 0 | 0 (0.0%) | 0 (0.0%) | 1 (10.0%) | 1 (2.7%) | 2 |
| HHV-6 | - | 0 (0.0%) | 0 (0.0%) | 0 (0.0%) | 0 | 3 (27.3%) | 0 (0.0%) | 0 (0.0%) | 3 (17.6%) | 6 |
| HSV-1 | 0 (0.0%) | 0 (0.0%) | 1 (10.0%) | 0 (0.0%) | 1 | 0 (0.0%) | 0 (0.0%) | 1 (6.3%) | 1 (2.1%) | 2 |
| ***Atypical bacteria*** | | | | | | | | | | |
| M.p. | 0 (0.0%) | 0 (0.0%) | 0 (0.0%) | 0 (0.0%) | 0 | 0 (0.0%) | 0 (0.0%) | 1 (6.3%) | 0 (0.0%) | 1 |

**c) Adults**:

|  | **Immunocompromised patients** | | | | | | **Immunocompetent patients** | | | | |
| --- | --- | --- | --- | --- | --- | --- | --- | --- | --- | --- | --- |
|  | **spring**  **(Mar-May)** | **summer**  **(Jun-Aug)** | **autumn**  **(Sep-Nov)** | **winter**  **(Dec –Feb)** | **total** | **spring**  **(Mar-May)** | | **summer**  **(Jun-Aug)** | **autumn**  **(Sep-Nov)** | **winter**  **(Dec –Feb)** | **total** |
| ***Respiratory viruses*** | | | | | | | | | | | |
| AdV | 2 (3.7%) | 0 (0.0%) | 1 (1.9%) | 0 (0.0%) | 3 | 0 (0.0%) | | 0 (0.0%) | 0 (0.0%) | 1 (1.5%) | 1 |
| BoV | 1 (1.9%) | 0 (0.0%) | 0 (0.0%) | 1 (2.0%) | 2 | 0 (0.0%) | | 0 (0.0%) | 0 (0.0%) | 1 (1.5%) | 1 |
| CoV | 2 (3.7%) | 0 (0.0%) | 0 (0.0%) | 2 (4.1%) | 4 | 1 (2.2%) | | 0 (0.0%) | 1 (2.9%) | 1 (1.5%) | 3 |
| EV | 1 (1.9%) | 0 (0.0%) | 1 (2.0%) | 0 (0.0%) | 2 | 0 (0.0%) | | 0 (0.0%) | 1 (2.9%) | 1 (1.5%) | 2 |
| HMPV | 0 (0.0%) | 0 (0.0%) | 0 (0.0%) | 2 (4.1%) | 2 | 2 (4.4%) | | 0 (0.0%) | 0 (0.0%) | 1 (1.5%) | 3 |
| HRV | 4 (7.4%) | 4 (12.1%) | 10 (19.2%) | 3 (6.1%) | 21 | 2 (4.4%) | | 2 (9.1%) | 5 (14.7%) | 4 (6.1%) | 13 |
| IV | 5 (9.3%) | 0 (0.0%) | 1 (1.9%) | 5 (10.2%) | 11 | 5 (11.1%) | | 0 (0.0%) | 5 (14.7%) | 18 (27.3%) | 28* |
| PiV | 2 (3.7%) | 1 (3.0%) | 2 (3.8%) | 1 (2.0%) | 6 | 2 (4.4%) | | 0 (0.0%) | 1 (2.9%) | 0 (0.0%) | 3 |
| RSV | 5 (9.3%) | 1 (3.0%) | 0 (0.0%) | 6 (12.2%) | 12* | 0 (0.0%) | | 0 (0.0%) | 0 (0.0%) | 4 (6.1%) | 4 |
| ***Herpesviruses*** | | | | | | | | | | | |
| CMV | 8 (14.8%) | 7 (21.2%) | 14 (26.9%) | 15 (30.6%) | 44 | 3 (6.7%) | | 2 (9.1%) | 2 (5.9%) | 3 (4.6%) | 10 |
| EBV | 17 (35.4%) | 9 (31.0%) | 14 (31.1%) | 15 (37.5%) | 55 | 8 (22.9%) | | 6 (31.6%) | 3 (15.8%) | 10 (18.9%) | 27 |
| HHV-6 | 5 (38.5%) | 3 (23.1%) | 5 (21.7%) | 2 (11.8%) | 15 | 3 (25.0%) | | 1 (9.1%) | 0 (0.0%) | 7 (41.2%) | 11* |
| HSV-1 | 13 (24.1%) | 5 (15.2%) | 4 (7.7%) | 9 (18.4%) | 31 | 6 (13.3%) | | 3 (13.6%) | 1 (2.9%) | 7 (10.6%) | 17 |
| VZV | 0 (0.0%) | 0 (0.0%) | 0 (0.0%) | 0 (0.0%) | 0 | 0 (0.0%) | | 0 (0.0%) | 0 (0.0%) | 1 (2.9%) | 1 |
| ***Atypical bacteria*** | | | | | | | | | | | |
| M.p. | 0 (0.0%) | 0 (0.0%) | 0 (0.0%) | 1 (2.0%) | 1 | 5 (11.1%) | | 3 (13.6%) | 5 (14.7%) | 3 (4.5%) | 16 |

* level of significance up to 5%

** level of significance up to 1%

*** level of significance up to ≤ 0.1

**Supplementary Figure 1:** Frequency of multiple pathogen detections observed in the overall study group (A) as well as in immunocompromised (B) and immunocompetent children (Ch) and adults (Ad) (C).

1. **Overall study group**

1. **Immunocompromised patients**

1. **Immunocompetent patients**

**Abbreviations:** AdV, adenovirus; BoV, bocavirus; CMV, cytomegalovirus; CoV, coronavirus; C.p., Chlamydia pneumoniae; EBV, Epstein-barr virus; EV, enterovirus; HHV-6, human herpesvirus 6; HMPV, human metapneumovirus; HPeV, human parechovirus; HRV, human rhinovirus; HSV-1, herpes simplex virus 1; HSV-2, herpes simplex virus 2; M.p., Mycoplasma pneumoniae; IV, influenzavirus; PiV, parainfluenzavirus; RSV, respiratory syncytial Virus; VZV, varicella-zoster virus
